# Increased Risk of Second Primary Cancers Following Diagnosis of Malignant Intraductal Papillary Mucinous Neoplasms of the Pancreas: A Population-Based Study

**DOI:** 10.3389/fonc.2019.00610

**Published:** 2019-07-09

**Authors:** Xiaoyi Huang, Bingbing Zhang, Jian Zhao, Chen Sun, Kaiwen Kong, Lulu Deng, Yanfang Liu, Jianming Zheng

**Affiliations:** ^1^Department of Pathology, Changhai Hospital, Second Military Medical University, Shanghai, China; ^2^Department of Orthopaedics, Changhai Hospital, Second Military Medical University, Shanghai, China

**Keywords:** IPMN, pancreas, SEER, follow-up, second primary cancer

## Abstract

**Introduction:** Several studies have reported that intraductal papillary mucinous neoplasms (IPMNs) of the pancreas are associated with extra-pancreatic malignancies. However, there have been no population-based studies evaluating the risk of second primary cancers (SPCs) in patients with pancreatic IPMN.

**Methods:** The Surveillance, Epidemiology, and End Results (SEER) database was used to identify and characterize data from patients with IPMN of the pancreas. The standard incidence ratio (SIR) of this cancer was calculated by estimating the relative risk (RR). A multivariate Cox regression model was used to estimate hazards ratios (HRs) of death and associated 95% CIs.

**Results:** Of 2,850 patients with IPMN of the pancreas, 104 patients (3.65%) developed 118 SPCs. The SIR for all SPCs combined was 1.22 (95% confidence interval [CI] = 1.01–1.46; *P* < 0.05). There was an elevated risk of site-specific SPCs in the small intestine (SIR = 8.68; 95% CI = 2.36–22.22), pancreas (SIR = 2.66; 95% CI = 1.15–5.25), urinary bladder (SIR = 2.02; 95% CI = 1.05–3.54), and eye and orbit (SIR = 13.47; 95% CI = 1.63–48.67) in patients with pancreas IPMN. In age subgrouping, people aged younger than 50 years had an increased risk of all-site SPC with an SIR of 6.44 (95% CI = 2.78–12.68). Cox regression modeling showed that advanced disease stage and a short latency period carried a higher risk of death in IPMN patients with SPC.

**Conclusions:** Patients diagnosed with pancreatic IPMNs were at higher risk than the general population for developing a second primary malignancy. Meanwhile, advanced historic stage and short latency period were associated with an elevated HR in IPMN patients who develop an SPC.

## Introduction

Intraductal papillary mucinous neoplasm (IPMN) of the pancreas is defined as a tumor producing mucus in the main pancreatic duct or side branches. Histologically, IPMN encompasses four grades of lesions: adenoma, borderline, carcinoma *in situ*, and invasive carcinoma (1). It is widely believed that IPMNs have malignant potential, following the typical pattern of adenoma-to-carcinoma (2). There are many factors that influence the development of IPMN into malignant pancreatic cancer, such as size, grade of dysplasia, and histological type (3, 4). Previous studies have demonstrated that progress from early IPMN to advanced IPMN is associated with an accumulation of genetic changes, which lead to pancreatic cancer (5). Due to their malignant potential, IPMNs require long-term observation and surgery is suggested in almost all cases (6). However, compared to pancreatic ductal adenocarcinoma, IPMNs have a more favorable prognosis (7).

Remarkably, several studies have described a phenomenon in which patients with IPMN have a higher risk of developing further organ cancers, compared to patients with other pancreatic diseases (1, 8, 9). According to a study conducted by Riall and colleagues, the incidence of additional primary malignancies in patients with invasive IPMN prior to and after surgery is about 10%. Further, their results suggested that 86% of additional primary cancers occurred before the diagnosis of pancreatic IPMN (1), while the risk of second primary cancers (SPCs) following diagnosis of malignant IPMN of the pancreas remains unclear. As our understanding of IPMN has improved and diagnostic scrutiny has increased over the past decade, the incidence and prognosis of IPMN have changed significantly. Recently, Baiocchi and colleagues conducted a review of the literature and found the incidence of SPC after diagnosis of IPMN had heterogeneity of 5–52% (10).

The objective of the current study was to evaluate the incidence of SPC in a large population-based cohort using the Surveillance, Epidemiology, and End Results (SEER) database (2000–2015). We calculated the standardized incidence ratio (SIR) of SPC after diagnosis of pancreatic IPMN between January 2000 and December 2015. The incidence of SPCs stratified by age and race were also analyzed. Additionally, multivariate Cox regression modeling was applied to assess the risk factors associated with SPC.

## Materials and Methods

The SEER Program of the National Cancer Institute collects and publishes data on cancer incidence and survival sourced from throughout the United States. In the present study, we employed data from the SEER 18 Regs dataset, excluding AK Research Data, Nov 2017 Sub (2000–2015) <Katrina/Rita Adjustment> (registries included: San Francisco-Oakland SMSA, Connecticut, Detroit, Hawaii, Iowa, New Mexico, Seattle, Utah, Atlanta, San Jose-Monterey, Los Angeles, Rural Georgia, California excluding SF/SJM/LA, Kentucky, Louisiana, New Jersey and Greater Georgia). This dataset was released April 2018 and is based on a November 2017 submission (11). This cohort provides the largest geographic coverage available, which covers approximately 27.8% of the U.S. population (based on the 2010 census). The SEER database is available to the public and all patient identities are protected. Our study was therefore exempted from institutional review board at our hospital.

We included all patients in the database who were diagnosed with IPMN of the pancreas between January 2000 and December 2015 according to the following criteria: site recode ICD-O-3/WHO 2008 = “Pancreas;” histologic type ICD-O-3 = “8050, 8260, 8450, 8453, 8471, 8480, 8481, 8503”. IPMN with carcinoma *in situ* or invasive carcinoma were included in our study, as benign neoplasms are not registered in the SEER database. We excluded death certificate and autopsy cases. A latency period of 6 months (the period from the time of primary IPMN diagnosis to the time of an SPC diagnosis) was required to reduce the possibility of synchronous primary cancers.

### Statistical Analyses

The SIR was estimated by calculating the relative risk (RR) obtained by dividing the number of observed SPCs from the SEER database by the expected number in the US general population from the SEER data publication. Besides, the excess risk (ER) per 10,000 person-years was calculated by dividing the difference between observed and expected number of cases by the number of person-years per risk and multiplied by 10,000 (12). In addition, separate subgroup analyses were conducted, stratified by age at IPMN diagnosis (<50 years, 50–65 years and >65 years), race (White, Black and Asian/Pacific Islander), and latency (6–11 months, 12–59 months, 60–120 months and >120 months). To determine whether the clinical characteristics of patients with IPMN had an independent effect on survival, we used a multivariate Cox regression model to estimate hazards ratios (HRs) and the associated 95% CI. Cox regression modeling included age at diagnosis of IPMN of the pancreas, race, gender, the behavior of IPMN, and latency time between the IPMN and the SPC. SEER^*^Stat 8.3.5 software was employed for statistical analysis. All *P*-values were two-sided and employed a significance level of 0.05.

## Results

A total of 2,850 patients diagnosed with IPMN and carcinoma *in situ* or invasive carcinoma were identified, with a total of 5988.20 person-years at risk. Among them, 104 patients with IPMN developed 118 SPCs among various sites (including hematopoietic diseases). Among this patient group, there were 70 men and 34 women with a mean age at diagnosis of 65.07 years and the median follow-up period was 76.74 years (range 5–188). The total observation period was 5988.20 person-years. The clinical characteristics of SPC patients are shown in Table 1.

**Table 1:** Clinical characteristics of patients with IPMN.

	**Person at risk = 2,850**	
	**Case number (*n*)**	**%**
Mean age, year (±SD)	65.07 (±10.11)	
Gender (total)	104	100%
Male	70	67.31%
Female	34	32.69%
Ethnicity	104	100%
White	89	85.58%
Black	11	10.58%
Asian or Pacific Islander	4	3.85%
Location of IPMN	104	
Head and neck	56	53.85%
Body and tail	19	18.27%
Other	29	27.88%
Size of IPMNs, mm (range)		
<10	2	1.92%
10–20	17	16.35%
21–30	9	8.65%
>30	47	45.19%
blank	29	27.88%
SEER stage		
Localized (and *in situ*)	67	64.42%
Regional	27	25.96%
Distant	9	8.65%
Unstaged	1	0.96%
Radiation therapy		
Yes	17	16.35%
None/Unknown	87	83.65%
Chemotherapy		
Yes	25	24.04%
None/Unknown	79	75.96%
Marital status		
Married	70	67.31%
Widowed	5	4.81%
Never married	17	16.35%
Divorced/Separated	11	10.58%
Unknown	1	0.96%
Median follow-up, mo (range)		76.74 (5–188)

*IPMN, intraductal papillary mucinous neoplasm; SD, Standard Deviation*.

Overall, there was an elevated risk of SPC at all sites (SIR = 1.22, 95% confidence interval [CI] = 1.01–1.46), compared with the general population. The most common site was the prostate (15.25%), lung and bronchus (14.41%), urinary bladder (10.17%), colon and rectum (6.78%), pancreas (6.78%) and breast (6.78%). In all solid tumors the SIR was 1.21 (95% CI = 1.00–1.45). As for specific sites, the risk was increased for the tongue (SIR = 5.97, 95% CI = 1.63–15.28), small intestine (SIR = 8.68, 95% CI = 2.36–22.22), pancreas (SIR = 2.66, 95% CI = 1.15–5.25), urinary bladder (SIR = 2.02, 95% CI = 1.05–3.54), as well as eye and orbit (SIR = 13.47, 95% CI = 1.63–48.67), compared with the general population. The result of SPC occurrence following pancreatic IPMN is displayed in Table 2.

**Table 2:** SPC occurrence following pancreatic IPMN.

	**Total**				
	**Person at risk = 2,850; Person year at risk = 5988.20**				
**Site**	**O**	**E**	**SIR**	**95% CI**	**ER**
All sites	118	97.04	1.22	1.01–1.46	35.01
All solid tumors	117	96.57	1.21	1.00–1.45	34.11
Oral cavity and pharynx	5	2.27	2.20	0.72–5.14	4.56
Tongue	4	0.67	5.97	1.63–15.28	5.56
Pharynx	1	0.33	2.99	0.08–16.67	1.11
Oropharynx	1	0.09	11.45	0.29–63.81	1.52
Digestive System	27	19.14	1.41	0.93–2.05	13.13
Stomach	2	1.79	1.12	0.14–4.03	0.35
Small Intestine	4	0.46	8.68	2.36–22.22	5.91
Colon, Rectum and Anus	9	9.84	0.91	0.42–1.74	−1.40
Liver and Gallbladder, Bile duct and other Biliary	4	2.85	1.56	0.42–3.97	2.37
Pancreas	8	3.00	2.66	1.15–5.25	8.34
Respiratory system	18	16.00	1.12	0.67–1.78	3.33
Larynx	1	0.80	1.26	0.03–7.00	0.34
Lung and bronchus	17	15.06	1.13	0.66–1.81	3.24
Skin excluding basal and squamous	7	4.81	1.46	0.59–3.00	3.66
Melanoma of the skin	6	4.34	1.38	0.51–3.01	2.77
Other non-epithelial skin	1	0.47	2.15	0.05–11.96	0.89
Breast	8	9.66	0.83	0.36–1.63	−2.76
Female genital system	2	3.69	0.54	0.07–1.96	−2.82
Corpus and uterus, NOS	1	2.03	0.49	0.01–2.74	−1.73
Ovary	1	0.96	1.05	0.03–5.83	0.07
Male genital system	18	17.83	1.01	0.60–1.60	0.28
Prostate	18	17.62	1.02	0.61–1.61	0.63
Urinary system	16	9.42	1.70	0.97–2.76	10.99
Urinary bladder	12	5.93	2.02	1.05–3.54	10.14
Kidney and renal pelvis	3	3.24	0.93	0.19–2.71	−0.39
Renal pelvis, ureter and other Urinary organs	2	0.51	3.94	0.48–14.23	2.49
Eye and orbit	2	0.15	13.47	1.63–48.67	3.09
Eye and orbit - non–melanoma	1	0.03	30.40	0.77–169.38	1.62
Eye and orbit - melanoma	1	0.12	8.66	0.22–48.23	1.48
Brain and other nervous system	1	0.97	1.03	0.03–5.73	0.05
Cranial nerves other nervous system	1	0.04	25.31	0.64–141.03	1.60
Endocrine system	3	1.32	2.27	0.47–6.63	2.80
Thyroid	3	1.23	2.44	0.50–7.14	2.96
All lymphatic and hematopoietic diseases	9	8.71	1.03	0.47–1.96	0.48
Lymphoma	5	4.40	1.14	0.37–2.65	1.00
Non-Hodgkin lymphoma	5	4.19	1.19	0.39–2.79	1.36
Myeloma	3	1.54	1.95	0.40–5.70	2.44
Leukemia	1	2.77	0.36	0.01–2.01	−2.96
Miscellaneous	2	2.08	0.96	0.12–3.47	−0.14

### Risk of SPC Stratified by Age at Diagnosis

We examined whether age at IPMN diagnosis affected risk for SPC. Age at diagnosis was classified as <50 years, 50–64 years, or >65 years. The risk of developing SPC at any site in patients with IPMN younger than 50 years was increased (SIR = 6.44, 95% CI = 2.78–12.78). SIR of site-specific risks were as follows: colon, rectum and anus (SIR = 18.41, 95% CI = 2.23–71.39), brain and other nervous system (SIR = 40.75, 95% CI = 1.03–227.03), lymphatic and hematopoietic diseases (SIR = 17.44, 95% CI = 2.11–63.00), and miscellaneous (SIR = 78.92, 95% CI = 2.00–439.72). In patients aged 50–64 years, there was no significant association between the risk of SPC and IPMN diagnosis at all sites. However, the risk of developing SPC in the tongue (SIR = 11.22, 95% CI = 1.36–40.53) and pancreas (SIR = 12.17, 95% CI = 3.95–28.40) were significantly increased. For patients older than 65 years, the risk of developing SPC at all sites was similar to the general population, while the risk of developing SPC at the small intestine increased significantly (SIR = 8.10, 1.67–23.66). The site-specific risk of SPC stratified by age at diagnosis is shown in Table 3.

**Table 3:** SPC occurrence following pancreatic IPMN stratified by age.

	**Observed**	**SIR**	**95% CI**	**ER**
<50 years				
All sites	8	6.44	2.78–12.68	116.46
All solid tumors	5	4.50	1.46–10.51	67.05
Digestive system	2	11.12	1.35–40.15	31.37
Colon, rectum, and anus	2	18.41	2.23–71.39	32.60
Female genital system	1	0.12	0.22–48.40	15.25
Brain and other nervous system	1	40.75	1.03–227.03	16.81
All lymphatic and hematopoietic diseases	2	17.44	2.11–63.00	32.49
Miscellaneous	1	78.92	2.00–439.72	17.02
50–64 years				
All sites	21	1.23	0.76–1.88	22.06
All solid tumors	20	1.29	0.79–1.99	25.40
Oral cavity and pharynx	2	3.63	0.44–13.11	8.24
Tongue	2	11.22	1.36–40.53	10.35
Digestive system	6	1.94	0.71–4.23	16.54
Pancreas	5	12.17	3.95–28.40	26.08
Respiratory system	1	0.46	0.01–2.56	−6.67
Lung and bronchus	1	0.51	0.01–2.82	−5.55
Melanoma of the skin	3	3.64	0.75–10.64	12.37
Breast	3	1.22	0.25–3.57	3.08
Male genital system	3	0.88	0.18–2.58	−2.27
Prostate	3	0.89	0.18–2.61	−2.02
Urinary system	2	1.57	0.19–5.65	4.11
Urinary bladder	2	3.28	0.40–11.84	7.90
All lymphatic and hematopoietic diseases	1	0.78	0.02–4.32	−1.65
Lymphoma	1	1.44	0.04–8.04	1.75
>65 years				
All sites	89	1.13	0.91–1.39	28.29
All solid tumors	81	1.17	0.93–1.46	32.52
Oral cavity and pharynx	3	1.78	0.37–5.22	3.62
Tongue	2	4.15	0.50–14.99	4.16
Pharynx	1	4.11	(0.10–22.91	2.07
Digestive system	19	1.20	0.72–1.87	8.58
Stomach	2	1.31	0.16–4.72	1.29
Small intestine	3	8.10	1.67–23.66	7.21
Colon, rectum, and anus	7	0.86	0.34–1.77	−3.18
Liver	3	2.48	0.51–7.25	4.91
Intrahep bile duct	1	5.74	0.15–31.96	2.26
Pancreas	3	1.16	0.24–3.40	1.16
Respiratory system	17	1.24	0.72–1.98	8.88
Larynx	1	1.61	0.04–8.95	1.03
Lung and bronchus	16	1.23	0.70–2.00	8.21
Skin excluding basal and squamous	4	1.03	0.28–2.63	0.30
Breast	5	0.73	0.24–1.70	−5.12
Female genital system	1	0.39	0.01–2.18	−4.25
Corpus uteri	1	0.76	0.02–4.22	−0.88
Male genital system	15	1.04	0.58–1.72	1.74
Prostate	15	1.05	0.59–1.74	2.13
Urinary system	14	1.73	0.95–2.91	16.24
Urinary bladder	10	1.89	0.91–3.47	12.89
Kidney and renal pelvis	3	1.18	0.24–3.45	1.25
Eye and orbit	2	16.95	2.05–61.22	5.16
Endocrine system	2	2.47	0.30–8.94	3.27
Thyroid	2	2.71	0.33–9.80	3.46
All lymphatic and hematopoietic diseases	6	0.82	0.30–1.79	−3.59
Lymphoma	3	0.82	0.17–2.41	−1.75
Myeloma	3	2.30	0.47–6.73	4.65
Miscellaneous	1	0.55	0.01–3.06	−2.25

### Risk of SPC Stratified by Race

In the patient cohort who developed a SPC, 89 (85.58%) were White, 11 (10.58%) were Black, and 4 (3.85%) were Asian or Pacific Islander. Among White patients, the SIR of developing SPC in all sites was 1.25 (95% CI = 1.02–1.52), and the SIR of developing SPC in all solid tumors was similar (SIR = 1.30, 95% CI = 1.02–1.52). Caucasians had a significantly higher risk of SPCs of the tongue (SIR = 6.77, 95% CI = 1.84–17.32), and urinary bladder (SIR = 2.23, 95% CI = 1.15–3.89). In the Black racial subgroup, there was no significant change in the risk of developing an SPC at all sites (SIR = 1.55, 95% CI = 0.80–2.71). However, the risk of developing an SPC increased in the oropharynx (SIR = 113.31, 95% CI = 2.87–631.32), small intestine (SIR = 34.04, 95% CI = 4.12–122.95), and myeloma (SIR = 13.6, 95% CI = 2.71–38.46). In the Asian or Pacific Islander subgroup, the risk of developing an SPC at all sites was not significantly changed (SIR = 0.57, 95% CI = 0.16–1.46), compared with the general population but their risk of SPCs in eye and orbit was increased (SIR = 259, 95% CI = 6.56–1443.07). The result is shown in Table 4.

**Table 4:** SPC occurrence following pancreatic IPMN stratified by race.

	**Observed**	**SIR**	**95% CI**	**AER**
White				
All Sites	102	1.25	1.02–1.52	44.09
All solid tumors	93	1.30	1.02–1.52	42.88
Oral cavity and pharynx	4	2.04	0.56–5.23	4.36
Tongue	4	6.77	1.84–17.32	7.28
Digestive system	21	1.39	0.86–2.12	12.50
Respiratory system	17	1.27	0.74–2.03	7.61
Skin excluding basal and squamous	7	1.49	0.60–3.07	4.93
Breast	0.93	0.93	0.37–1.91	−1.17
Female genital system	2	0.70	0.08–2.52	−1.85
Male genital system	15	0.98	0.55–1.62	−0.57
Prostate	15	0.99	0.56–1.64	−0.18
Urinary system	16	1.92	1.10–3.11	16.34
Urinary bladder	12	2.23	1.15–3.89	14.11
Eye and orbit	1	7.08	0.18–39.45	1.83
Brain and other nervous system	1	1.16	0.03–6.46	0.29
Endocrine system	3	2.84	0.58–8.29	4.15
All lymphoma and hematopoietic diseases	6	0.81	0.30–1.76	−3.06
Miscellaneous	2	1.15	0.14–4.16	0.58
Black				
All sites	12	1.55	0.80–2.71	80.65
All solid tumors	9	1.30	0.59–2.46	38.83
Oral cavity and pharynx	1	7.42	0.19–41.35	16.37
Oropharynx	1	113.31	2.87–631.32	18.76
Digestive system	5	2.85	0.93–6.66	61.45
Small intestine	2	34.04	4.12–122.95	36.73
Respiratory system	1	0.76	0.02–4.23	−6.03
Breast	1	0.91	0.02–5.09	−1.80
Male genital system	1	0.67	0.02–3.76	−9.14
Prostate	1	0.68	0.02–3.78	−8.95
All lymphoma and hematopoietic diseases	3	4.98	1.02–14.49	45.32
Myeloma	3	13.16	2.71–38.46	52.46
Asian or Pacific Islander				
All Sites	4	0.57	0.16–1.46	−43.91
All solid tumors	4	0.64	0.17–1.63	−32.98
Digestive system	1	0.49	0.01–2.74	−15.03
Pancreas	1	3.64	0.09–20.27	10.55
Male genital system	2	1.96	0.24–7.09	14.28
Prostate	2	1.99	0.24–7.18	14.48
Eye and orbit	1	259.00	6.56–1443.07	14.50

### Risk of SPC Stratified by Latency

The entire cohort was divided into four latency intervals: 6–11, 12–59, 60–119 months, and more than 120 months following the initial diagnosis of pancreatic IPMN. We observed a significant difference in risk of SPC at all sites at 60–119 months (SIR = 1.49, 95% CI = 1.03–2.08), and more than 120 months (SIR = 2.25, 95% CI = 1.08–4.14), compared to the general population. The risk of SPC for each latency period is shown in Table 5.

**Table 5:** SPC occurrence following pancreatic IPMN stratified by latency.

	**6–11 months**				**12–59 months**				**60–119 months**				**120+ months**			
	**O**	**SIR**	**95% CI**	**ER**	**O**	**SIR**	**95% CI**	**ER**	**O**	**SIR**	**95% CI**	**ER**	**O**	**SIR**	**95% CI**	**ER**
All sites	18	1.06	0.63–1.67	8.74	56	1.06	0.80–1.38	10.05	34	1.49	1.03–2.08	84.35	10	2.25	1.08–4.14	194.49
All solid tumors	16	1.06	0.60–1.72	7.79	48	1.03	0.76–1.36	3.92	32	1.60	1.09–2.25	90.51	10	2.58	1.24–4.75	214.28
Oral cavity and pharynx	1	2.53	0.06–125.67	−0.27	3	2.43	0.50–7.10	5.38	1	1.86	0.05–10.36	3.50	0			
Digestive system	3	0.90	0.19–2.63	−2.98	12	1.16	0.60–2.03	5.10	9	1.96	0.90–3.73	33.45	3	3.33	0.69–9.73	73.41
Respiratory system	0				7	0.81	0.32–1.68	−5.10	8	2.10	0.90–4.13	31.67	3	4.14	0.85–12.11	79.62
Breast	2	1.03	0.12–3.72	0.53	3	0.57	0.12–1.67	−6.90	2	1.01	0.12–3.66	0.19	1	2.10	0.05–11.87	18.57
Female genital System	1	1.35	0.03–7.51	2.34	1	0.50	0.01–2.76	−3.10	0				0			
Male genital system	7	2.24	0.90–4.62	35.20	8	0.80	0.34–1.57	−6.16	2	0.49	0.06–1.78	−15.57	1	1.59	0.04–8.84	12.94
Urinary system	1	0.65	0.02–3.65	−4.78	6	1.19	0.44–2.60	2.97	8	3.33	1.44–6.56	42.37	1	2.15	0.05–12.00	18.74
Brain and other nervous system	0				1	1.89	0.05–10.55	1.44	0				0			
endocrine system	1	4.27	0.11–23.80	6.95	2	2.77	0.34–10.00	3.89	0				0			
All Lymphatic and hematopoietic diseases	2	1.37	0.17–4.94	4.88	6	1.29	0.47–2.81	4.13	1	0.46	0.01–2.57	−8.88	0			

### Overall Survival and Clinical Characteristics

To determine the factors that may affect survival time in patients with SPC, Cox regression modeling was employed. Compared with the localized stage of IPMN, regional disease with the diagnosis of SPC appeared to have an increased risk of death (HR = 2.652, 95% CI = 1.207–5.828). Similarly, distant disease at the diagnosis of SPC appeared to have an increased risk of death (HR = 4.351, 95% CI = 1.633–11.591). As for latency time, shorter than 60 months appeared to have a high risk of death (HR = 2.95, 95% CI = 1.503–5.731). The HR of overall survival of IPMN patients with SPC is shown in Table 6.

**Table 6:** Overall survival of pancreatic IPMN patients with SPC.

**Variable**	**HR**	**95% CI**	***P***
Race			
White		Reference	
Black	0.809	0.236–2.779	0.737
Asian or Pacific Islander	0.545	0.127–2.350	0.416
Gender			
Male		Reference	
Female	0.805	0.455–1.424	0.457
Age			
<65 year		Reference	
≥65 year	0.726	0.402–1.312	0.288
Behavior			
*In situ*		Reference	
Malignant	0.849	0.449–1.606	0.615
SEER historic stage			
Localized		Reference	
Regional	2.652	1.207–5.828	0.015
Distant	4.351	1.633–11.591	0.003
Latency			
6–59 month	2.935	1.503–5.731	0.002
≥ 60 month		Reference	

*HR, hazards ratio; CI, confidence interval*.

## Discussion

There have been few studies focusing on malignancies following diagnosis of IPMN in the pancreas. To the best of our knowledge, the present study represents the largest cohort used to study the risk of developing SPC after IPMN of the pancreas. In total, 2,850 patients diagnosed between January 2000 and December 2015 with pancreatic IPMNs were identified from the SEER database. Among these patients, a total of 104 patients developed SPCs during the follow-up period. According to our data, after an initial diagnosis of pancreatic IPMN, all patients had an elevated risk of developing SPCs, especially in the tongue, small intestine, pancreas, urinary bladder, and eye and orbit.

Both tongue and small bowel cancers are rare in the general population. It is estimated that the incidence rate of tongue cancer is up to 6.5–8.0 persons per 100,000 persons per annum in Asia and Europe (13). Similarly, small bowel cancers are rare tumors with an incidence of 22.7 cases million in the United States (14). Although it is difficult to address the association between the elevated risk of tongue cancer and a diagnosis of IPMN, some previous studies suggest that gene mutation is the tongue is a cause of susceptibility. Similarly, Lee and colleagues showed that the prevalence of KRAS mutation in IPMN of the pancreas was about 60% (15). Das and colleagues reported that the frequency of mutation in the KRAS gene of oral cancer including tongue cancer was 33% (16). However, there have been no studies showing that these two tumors are directly related. Similarly the association between IPMN of the pancreas and bladder cancer have not been previously reported.

Non-invasive precursor lesions in the pancreas, including IPMN, mucinous cystic neoplasm (MCN) and pancreatic intraepithelial neoplasia (PanIN) are related to pancreatic cancer (17). The incidence of pancreatic cancer related to IPMN has been reported in many previous investigations and international consensus guidelines (18–20). Generally, IPMN is either of the branch duct (BD-IPMN) or the main duct (MD-IPMN) according to morphology. As summarized by Tanaka et al, the proportion of invasive carcinoma in MD-IPMN is ~40%, while the proportion of invasive carcinoma in BD-IPMN is relatively low at 13.1% (21). We should emphasize that since the SEER database does not include specific information on morphological types, we could not calculate the ratio of MD-IPMN and BD-IPMN.

In our study, an elevated risk of developing pancreatic cancer was observed, consistent with previous study (22). Kawakubo et al. conducted a cohort study of 642 patients with IPMNs and found that the incidence of pancreatic cancer was significantly higher in IPMN patients, compared with the general population (22). There are two main factors among IPMNs at high risk of pancreatic malignancy: molecular mutation, and preoperative work-up. Many studies showed that IPMN and pancreatic cancer have the same somatic mutations, such as in KRAS and GNAS (23, 24). KRAS mutations are the most common alterations in IPMN of the pancreas and are considered as an early event of pancreatic cancer development (25, 26). Mutations in GNAS are found in 40–70% of IPMNs and 20–40% of pancreatic invasive carcinomas (27–29). Mutations in other cancer-related genes, such as SMAD4, PI3KCA, PTEN, and BRAF, have been reported by previous studies in both IPMNs and pancreatic cancers with lower prevalence (30–33). Another consideration is the importance of comprehensive preoperative work-up. The management of IPMN changed from early resection to more deliberate surveillance (34). Because surgery is frequently performed to treat IPMN in the past two decades, these patients were more likely to receive comprehensive preoperative examination and postoperative follow-up, increasing the chances of finding pancreatic malignancy. However, currently available data cannot clarify the association between the elevated risk of pancreatic cancer and lack of clinical observation in patients with IPMN of the pancreas.

Some specific differences were observed when patients were stratified by age for risk of SPC. In the age subgroup of >50 years, the risk of developing SPC was significantly higher. In the 50–64 year subgroup, there was increased risk of the tongue and pancreatic cancer following IPMN. Contrastingly, the risk of small intestine and eye and orbit malignancy was elevated in the age subgroup of older than 65 years. When it came to racial subgrouping, White patients had an increased risk of developing SPC, while the risk of Black patients developing SPC was similar to the general population. It was therefore assumed that White race and advanced age were risk factors for SPC in IPMN patients, but there was no available data to determine the exact association (10). As for latency subgrouping, the risk of developing SPC was elevated in subgroups with a latency period of 60–119 months and more 120 months.

Multivariate Cox regression modeling revealed the negative influence of advanced historic stage and shorter latency time in the development of SPC and overall survival in IPMN patients. Compared with its localized stage, IPMN in regional and distant stage had an increased risk of death. There is no previous study that reports the risk of death for SPC of IPMN patients. In the current study, HR was higher for patients with a latency <5 years, compared with those whose latency more than 5 years. At present, there is no article fully reporting the long-term cause of death in patients with IPMN. Due to a lack of detailed information on the cause of death, we could not calculate the HR of cancer-specific survival to estimate the influence of SPC on IPMN patients. Thus, the result of overall survival should be interpreted with caution.

The major strength of the current study is the use of a large clinical dataset representing patient diversity from a wide geographical area, which ensures the generalizability of the results. However, some limitations should be noted. The SEER database lacks complete, detailed clinical information, such as treatment strategy such as dose of chemotherapy and radiation. Second, information on patient exposure to environmental factors including smoking, diet, and pollutants, which might influence the incidence of cancer and prognosis (35), are not provided by the SEER database. Finally, the numbers of SPC patients for some race and age groups with IPMN of the pancreas were relatively small, which may have led to selection bias.

## Conclusion

This large population-based study suggested that patients with IPMN of the pancreas have a higher risk of developing SPC than the general population. Among all sites of SPC, the tongue, small intestine, pancreas, bladder, and eye and orbit had an increased risk of developing SPC. Subgroup analysis showed that specific age, White race, and a long latency period may be risk factors related to the development of SPC. Meanwhile, advanced disease stage and short latency period are associated with high HR of death in IPMN patients who develop SPC. Clinicians treating IPMN of the pancreas should pay attention to the occurrence of SPC during follow-up. Further study is warranted to verify the results of current study.

## Ethics Statement

The SEER database is available to the public and all patient identities are protected. Our study was therefore exempted from institutional review board at our hospital.

## Author Contributions

XH, BZ, YL, and JZheng are lead authors who participated in data collection, manuscript drafting, table/figure creation, and manuscript revision. JZhao, CS, and KK aided in data collection. XH and BZ are senior authors who aided in drafting the manuscript and manuscript revision. YL and JZheng is the corresponding author who initially developed the concept and drafted and revised the manuscript. All authors read and approved the final manuscript.

### Conflict of Interest Statement

The authors declare that the research was conducted in the absence of any commercial or financial relationships that could be construed as a potential conflict of interest.
